# The Pathogenesis of Systemic Sclerosis: An Understanding Based on a Common Pathologic Cascade across Multiple Organs and Additional Organ-Specific Pathologies

**DOI:** 10.3390/jcm9092687

**Published:** 2020-08-19

**Authors:** Yoshihide Asano

**Affiliations:** Department of Dermatology, University of Tokyo Graduate School of Medicine, 7-3-1 Hongo, Bunkyo-ku, Tokyo 113-8655, Japan; asanoy-der@h.u-tokyo.ac.jp; Tel.: +81-3-3815-5411; Fax: +81-3-3814-1503

**Keywords:** systemic sclerosis, a common pathologic cascade across multiple organs, additional organ-specific pathologies

## Abstract

Systemic sclerosis (SSc) is a multisystem autoimmune and vascular disease resulting in fibrosis of various organs with unknown etiology. Accumulating evidence suggests that a common pathologic cascade across multiple organs and additional organ-specific pathologies underpin SSc development. The common pathologic cascade starts with vascular injury due to autoimmune attacks and unknown environmental factors. After that, dysregulated angiogenesis and defective vasculogenesis promote vascular structural abnormalities, such as capillary loss and arteriolar stenosis, while aberrantly activated endothelial cells facilitate the infiltration of circulating immune cells into perivascular areas of various organs. Arteriolar stenosis directly causes pulmonary arterial hypertension, scleroderma renal crisis and digital ulcers. Chronic inflammation persistently activates interstitial fibroblasts, leading to the irreversible fibrosis of multiple organs. The common pathologic cascade interacts with a variety of modifying factors in each organ, such as keratinocytes and adipocytes in the skin, esophageal stratified squamous epithelia and myenteric nerve system in gastrointestinal tract, vasospasm of arterioles in the heart and kidney, and microaspiration of gastric content in the lung. To better understand SSc pathogenesis and develop new disease-modifying therapies, it is quite important to understand the complex pathogenesis of SSc from the two distinct perspectives, namely the common pathologic cascade and additional organ-specific pathologies.

## 1. Introduction

Systemic sclerosis (SSc) is a multisystem connective tissue disease with unknown etiology, characterized by aberrant immune activation, vascular injury followed by defective neovascularization and impaired vessel remodeling, and resultant tissue fibrosis of the skin and various internal organs [1,2,3]. SSc presents with a variety of clinical symptoms, including skin sclerosis, digital ulcers, interstitial lung disease (ILD), pulmonary hypertension (PH), cardiac fibrosis, gastrointestinal (GI) involvement, liver dysfunction and scleroderma renal crisis (SRC), all of which are caused by a common disease-specific pathologic cascade across multiple organs and additional organ-specific pathologies. In terms of the management of SSc, it is quite important to understand “the common pathologic cascade across multiple organs” and “the additional organ-specific pathologies” separately. This article overviews the understanding of SSc pathogenesis based on these two distinct aspects.

## 2. The Common SSc-Specific Pathologic Cascade across Multiple Organs

**Figure 1:**
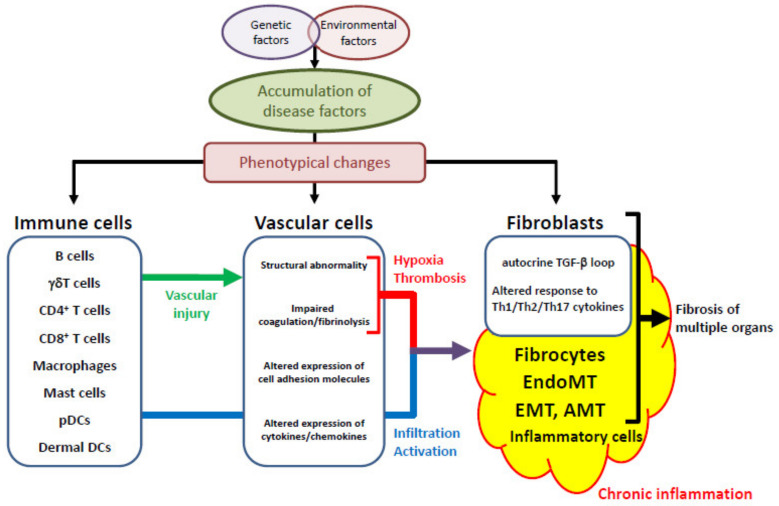
The SSc-specific common pathologic cascade across multiple organs. The accumulation of predisposing factors through the interaction of genetic factors and environmental influences induces the phenotypical alterations of immune cells, vascular cells and interstitial fibroblasts. Vascular injury due to autoimmune attacks and unknown environmental influences triggers the SSc-specific common pathologic cascade across multiple organs, starting with vascular activation and vascular structural abnormalities, and subsequently resulting in chronic inflammation and the related activation of interstitial fibroblasts derived from various sources. pDCs, plasmacytoid dendritic cells; DCs, dendritic cells; TGF-β, transforming growth factor-β; Th, T helper; EndoMT, endothelial-to-mesenchymal transition; EMT, epithelial-to-mesenchymal transition; AMT, adipocyte-to-myofibroblast transdifferentiation.

**Figure 2:**
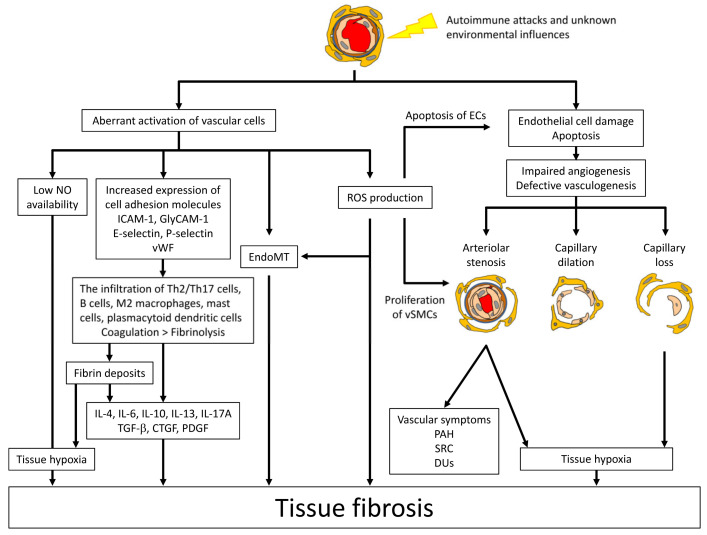
The role of vasculopathy bridging between immune abnormalities and fibrosis. Initial vascular injury is caused by autoimmune attacks and unknown environmental influences. Vascular injury results in structural and functional abnormalities in the vasculature characteristic of SSc. Structural abnormalities are classified into arteriolar stenosis, capillary dilation and capillary loss, which are attributable to impaired angiogenesis and defective vasculogenesis. The functional abnormalities include endothelial dysfunction, primarily due to the low availability of nitric oxide (NO), the altered expression of cell adhesion molecules inducing the infiltration of Th2 and Th17 cells, mast cells and macrophages, the activated endothelial-to-mesenchymal transition (EndoMT) leading to fibro-proliferative vascular change and tissue fibrosis, the impairing of the coagulation/fibrinolysis system promoting the formation of intravascular fibrin deposits, and the excessive production of reactive oxygen species (ROS). These vascular changes eventually induce the constitutive activation of interstitial fibroblasts in multiple organs. ICAM-1; intercellular cell adhesion molecule-1, GlyCAM-1; glycosylation-dependent cell adhesion molecule-1, vWF; von Willebrand factor, TGF-β; transforming growth factor-β, CTGF; connective tissue growth factor, PDGF; platelet-derived growth factor, ECs; endothelial cells, vSMCs; vascular smooth muscle cells, PAH; pulmonary arterial hypertension, DUs; digital ulcers, SRC; scleroderma renal crisis.

The common SSc-specific pathologic cascade across multiple organs is summarized in Figure 1. Vasculopathy is a critical pathological step bridging between autoimmunity and fibrosis (Figure 2). The details are described below.

### 2.1. Genetic Factors and Environmental Influences

Genetic and etiological studies have demonstrated that SSc is caused by a complex interplay between genetic factors and environmental influences. The highest risk factor of SSc is a family history [4], but concordance for SSc is low in twins (4.7%) and similar in monozygotic and dizygotic twins (4.2% versus 5.6%). Of note, concordance for the presence of autoantibodies against either nuclear or cytoplasmic antigens is significantly higher in the healthy monozygotic twin sibling than in the healthy dizygotic twin sibling of an SSc patient (95% versus 60%, *p* < 0.05) [5], suggesting that genetic factors appear to be linked to autoimmunity increasing the susceptibility to SSc, but not enough for the development of clinically definite SSc. In line with this idea, most of the susceptibility genes for SSc are Human Leukocyte Antigen (HLA) haplotypes and non-HLA genes related to immunity and inflammation, which are shared by other collagen diseases such as rheumatoid arthritis and systemic lupus erythematosus [6,7]. On the other hand, several case-control and genome-wide association studies show that the single nucleotide polymorphisms (SNPs) of certain disease-susceptibility genes correlate with disease severity of SSc [8,9,10,11,12,13]. For instance, a certain SNP related to the downregulation of interferon regulatory factor 5 (IRF5) is much more frequently detected in SSc patients with milder clinical symptoms [14]. Thus, genetic factors affect the susceptibility to and the severity of SSc.

Environmental and occupational factors relevant to SSc, including silica, solvents, epoxy resins and breast implants [15], potentially affect the behavior of various cell types by directly acting on cellular signaling pathways [16] and/or through epigenetic mechanisms [17]. Although still controversial in part, numerous clinical and experimental studies have revealed the critical contribution of environmental factors to SSc development, and occupational SSc is already an established clinical entity [15]. Taken together with the results of the twin study [5], it is speculated that certain set of environmental factors trigger the development of SSc in genetically predisposed individuals.

### 2.2. Vascular Injury, an Initial Event of SSc Development

Since the majority of SSc genetic factors are related to immunity and inflammation, the aberrant activation of the immune system after exposure to certain environmental influences seems to be the first step of the SSc disease process. Consistent with this notion, disease-specific autoantibodies are already present prior to the emergence of the first clinical symptoms associated with SSc, such as Raynaud’s phenomenon, puffy fingers and morning stiffness [18]. As represented by the usefulness of nailfold capillary changes for the early diagnosis of SSc [19], the initial target of autoimmune attacks is believed to be endothelial cells of small blood vessels, including capillaries and arterioles. So far, in vitro experiments with clinical samples have suggested that anti-endothelial cell antibodies and γδT cells comprise a part of the immunological aspects causing initial vascular injury [20,21,22,23,24], but other immune cells and environmental factors are also thought to be involved in this process [25,26,27]. At this moment, the detailed mechanism of initial vascular injury remains unknown due to the difficulty in obtaining skin biopsies and other clinical samples prior to the onset of the first SSc-related clinical manifestations.

### 2.3. Aberrant Vascular Reaction to Initial Vascular Injury

Endothelial cells attacked by autoimmunity and/or environmental factors are believed to undergo two distinct fates; cell death and cell activation. Injured blood vessels are abnormally repaired because neovascularization and vascular remodeling are extensively impaired, leading to the development of SSc-specific structural changes of small vessels. On the other hand, activated endothelial cells function abnormally, promoting inflammation and tissue fibrosis.

Neovascularization and vascular remodeling are basically composed of two distinct processes: angiogenesis and vasculogenesis. Angiogenesis is neovessel formation mediated by the proliferation and migration of pre-existing endothelial cells, while vasculogenesis is de novo vessel formation by the incorporation, differentiation, migration and/or proliferation of bone marrow-derived progenitor cells. Evidence suggests that dysregulated angiogenesis (imbalance of pro-angiogenic and anti-angiogenic factors) and defective vasculogenesis (decreased number, dysfunction and/or impaired recruitment of circulating bone marrow-derived progenitor cells) underpin the complex vascular pathology of SSc [28,29,30,31,32], resulting in the development of vascular structural alterations, such as capillary dilation, capillary loss and arteriolar stenosis.

Some of the attacked SSc endothelial cells overexpress cell adhesion molecules, chemokines, cytokines and growth factors, enhancing the interaction with circulating cells such as leukocytes and platelets and increasing the tethering, rolling, firm adhesion, extravasation and tissue infiltration of leukocytes. Generally, intercellular adhesion molecule-1 (ICAM-1) and glycosylation-dependent cell adhesion molecule-1 (GlyCAM-1, a physiologic ligand for L-selectin) on endothelial cells regulate the accumulation of Th2/Th17 cells, macrophages and mast cells, while E-selectin and P-selectin on endothelial cells regulate Th1 cell infiltration [33]. The lesional skin of early diffuse cutaneous SSc (dcSSc) highly expresses all of these cell adhesion molecules, especially ICAM-1 and GlyCAM-1 predominating over E-selectin and P-selectin [34]. Therefore, SSc endothelial cells theoretically possess the property of promoting the infiltration of Th2 and Th17 cells, macrophages and mast cells. Indeed, the infiltration of mast cells and macrophages is increased and the expression of Th2/Th17 cytokines is enhanced in SSc-involved skin during the early and progressive stage [35,36,37,38]. On the other hand, SSc peripheral blood mononuclear cells up-regulate cell adhesion molecules, targeting themselves to the endothelium [39]. These molecules include L-selectin and integrin α6—key leukocyte adhesion molecules involved in the initial tethering to the endothelial cells—and selectin P ligand regulating leukocyte rolling on endothelial cells and T cell skin homing, suggesting the high ability of SSc inflammatory cells to infiltrate into injured tissues.

Another feature of SSc vasculopathy is vascular disintegrity with the constitutively activated pro-angiogenic gene program at the molecular level, leading to the dilation of capillaries and the related leakage of red blood cells (nailfold bleeding) [29]. Furthermore, endothelial-to-mesenchymal transition (EndoMT) occurs in vasculature associated with tissue fibrosis, as shown in animal models of fibrotic disorders, such as diabetic nephropathy, cardiac fibrosis and bleomycin-induced dermal and pulmonary fibrosis [34,40,41,42,43]. Since EndoMT provides mesenchymal cells, which potentially participate in fibro-proliferative vasculopathy and tissue fibrosis, EndoMT is believed to be involved in the pathological process of SSc [44,45]. This notion has been confirmed recently with clinical samples derived from the skin and lungs of SSc patients [46,47].

In addition, SSc endothelial cells induce platelet activation and related intravascular fibrin deposition, leading to luminal narrowing and vessel obstruction. The increased production of vasoconstrictors such as endothelin, the underproduction of vasodilators such as prostacyclin and nitric oxygen, the increased expression of von Willebrand factor, and the decreased expression of endothelial protein C receptor may contribute to hypercoagulation status in SSc [48,49,50,51]. Furthermore, integrin αIIb and glycoprotein Ibβ, receptors for von Willebrand factor expressing on platelets, are abnormally up-regulated in SSc [39]. Given that sensitive markers of fibrinolytic enhancement, such as plasma levels of D-dimer and plasmin-α2-plasmin inhibitor complex (PIC), are elevated in SSc patients [51,52,53,54], the altered balance of coagulation/fibrinolysis system appears to cause a variable degree of luminal thrombosis following vascular injury, contributing to impaired peripheral circulation, the induction of inflammation and the subsequent activation of vascular cells (endothelial cells and pericytes/vascular smooth muscle cells) and fibroblasts [55,56,57].

### 2.4. Inflammation

As described above, the interaction of endothelial cells with circulating immune cells through cell adhesion molecules and chemokine promotes the activation of inflammatory cells and their infiltration into the affected organs of SSc. Generally, the infiltration of T cells, macrophages and mast cells is predominant, while B cell infiltration is relatively limited in SSc-involved skin [35,36,58,59]. On the other hand, numerous lymphoid aggregates composed of a large amount of B cells and relatively small amounts of T cells and macrophages are generally evident in the lung tissue of SSc-ILD [60]. Of note, activated B cell-related genes are up-regulated in SSc-involved skin [61], and B cell count in the skin correlates with the progression of skin fibrosis [59]. Thus, there is more or less difference in the types of infiltrating cells in each of the involved organs, but the infiltration of B cells, T cells and innate immune cells is commonly increased in the involved organs of SSc patients.

The alteration of T cell subsets has been well studied in SSc. The balances of Th1/Th2 and Th17/Treg immune responses skew toward Th2 and Th17 predominance [37,38,62,63], and Treg function is impaired in the active stage of SSc [64]. In the early stage of dcSSc, serum IL-6 and IL-10 levels are significantly elevated, while they are decreased to normal levels in the late stage of dcSSc, characterized by the regression of skin sclerosis [65]. IL-4 is maintained at normal levels in the early stage of dcSSc, but is decreased along with the resolution of skin sclerosis. In contrast, serum IL-12 levels are decreased in the early stage of dcSSc, then gradually increased in parallel with disease duration, and finally reach significantly higher levels than normal controls in the late stage of dcSSc [37]. As for Th17 cytokines, the expression levels of IL-17A, IL-21 and IL-22, but not of IL-17F, are increased in the lesional skin of early dcSSc [63,66]. Furthermore, the percentages of circulating Th17 cells and IL-17 production are elevated in SSc peripheral blood mononuclear cells, and the number of Th17 cells correlates with disease activity [38]. With regard to Tregs, the proportion of Th2-like Tregs is increased in the involved skin of SSc patients [58].

At this moment, the direct role of SSc-related antinuclear antibodies, such as antibodies against topoisomerase I (topo I), centromere and RNA polymerase III (RNAP III) antigens, remains unknown, although a potential role of anti-topoisomerase I antibody is proposed (described below). However, the close association of these antibodies with clinical manifestations suggests that altered B cell phenotypes possibly correlate with the central abnormality driving the progression of this disease through the genetic and epigenetic mechanisms shared with other cell types, and/or the complex interaction with other immune and non-immune cells. In addition to antibody production, B cells play multifaceted roles in the immune system, such as cytokine production, antigen presentation, macrophage differentiation and activation, and lymphoid tissue development [67]. SSc B cells are constitutively activated, as represented by the up-regulated expression of CD19, an accelerator of B cells [68], and the up-regulated expression of CD80 and CD86 on the memory B cells [69]. Supporting the significance of B cells in the SSc-specific disease cascade, B cell depletion therapy with rituximab, an anti-CD20 antibody, improved skin fibrosis and ILD in several case series and open studies [70,71,72,73,74,75]. In addition, there are several case reports or case series in which calcinosis, digital ulcers, or arterial stiffness were improved by rituximab therapy [76,77,78]. Thus, B cells are involved in the activation of vascular and fibrotic processes in addition to the activation of the immune system in SSc, supporting the idea that immune cells are located in the upstream of the SSc-specific disease cascade.

In addition to adaptive immune cells, innate immune cells abundantly infiltrate into SSc-involved organs. In SSc lesional skin, mast cells secrete an excessive amount of transforming growth factor (TGF)-β [79]. Furthermore, M2 macrophages seem to be a critical regulator of tissue fibrosis because the M2 macrophage-related gene program, which is up-regulated in the skin of early SSc patients, is suppressed in parallel with the resolution of skin fibrosis by the treatment with tocilizumab (an anti-IL-6 receptor antibody) [80].

Plasmacytoid dendritic cells (pDCs) produce a large amount of interferon (IFN)-α through the activation of Toll-like receptor (TLR) 7 and 9. In SSc-involved skin, the expression of chemerin, a potent chemoattractant for pDCs, is increased in dermal small vessels, and pDCs are distributed around small vessels [25,26]. In addition, the expression of LL-37, which interacts with self-DNA and facilitates its conversion into a stimulatory ligand for TLR7 and 9, is increased in small dermal vessels [27]. Given that endothelial death due to autoimmune attacks provides self-DNA, it is assumed that pDCs produce excessive amounts of IFN-α around small dermal vessels through the activation of TLR7 and 9 via the complex of self-DNA and LL-37 derived from endothelial cells. As well as this, disease-associated autoantibodies, especially anti-topoisomerase I antibody, potentially contribute to this process. Anti-topoisomerase I antibody reacts with nuclear antigens derived from endothelial cells, and the immune complex with nucleic acids, in particular RNA, induces IFN-α production from pDCs [81]. Indeed, previous clinical observations and experimental studies support the notion that IFN-α promotes the development and progression of SSc. For instance, in the clinical trial of recombinant IFN-α for SSc patients, withdrawal from the protocol occurred more frequently in SSc patients treated with IFN-α than in those treated with placebo, and most patients who dropped out of IFN-α treatment experienced the exacerbation of ILD [82]. Furthermore, IFN-α treatment for other diseases, such as multiple sclerosis and chronic hepatitis C virus infection, induces the onset of typical SSc or SSc-like symptoms [83,84,85,86,87,88]. Given that continuous exposure to IFN-α induces the senescence of endothelial cells [89], possibly amplifying IFN-α production from pDCs by providing self-DNA, endothelial death and pDCs form a feedforward loop promoting the progression of SSc through vascular injury and the activation of the immune system by IFN-α. Of interest, a recent study has demonstrated the ectopic expression of TLR8 in SSc pDCs, and a pivotal role of TLR8-expressing pDCs in experimental skin fibrosis, suggesting that TLR8 is the key RNA-sensing TLR, and that the overproduction of IFN-α is involved in the establishment of SSc-associated tissue fibrosis [90]. Overall, the continuous release of autoantigens from injured and senescent endothelial cells is a fundamental fuel of SSc pathology, which acts by inducing chronic inflammation.

### 2.5. The Activation of Fibroblasts

The activation of fibroblasts is the final consequence of the SSc-specific disease cascade. In the involved organs, there are lots of α-SMA-positive myofibroblasts, which produce excessive amounts of extracellular matrix (ECM). These myofibroblasts originate from resident fibroblasts, bone marrow-derived fibrocytes [91], epithelial-to-mesenchymal transition [92], EndoMT [46,47] and adipocyte-to-myofibroblast transdifferentiation [93]. These cells enact both intrinsic and extrinsic mechanisms to keep the constitutively activated status during disease progression.

A key growth factor involved in the activation of SSc dermal fibroblasts is TGF-β, a potent inducer of ECM including fibrillar collagens constituting the dermis (type I, III and V). TGF-β expression in the involved skin is elevated in patients with early active disease, but is weak or undetectable in those with established fibrosis. The expression profile of the three isoforms of TGF-β is recognized as follows in SSc: (i) all the three isoforms of TGF-β are detectable in ECM, and (ii) the expression of TGF-β1 and TGF-β2 is most prominent around dermal vessels, and is associated with perivascular infiltrating mononuclear cells [94,95,96]. In the early stages of SSc, therefore, TGF-β appears to promote inflammation by recruiting leukocytes through the regulation of cell adhesion molecules and the generation of chemokine gradient, by activating leukocytes, and by inducing various proinflammatory cytokines and other mediators. On the other hand, in the sclerotic phase SSc dermal fibroblasts are constitutively activated with a pro-fibrotic phenotype similar to that of normal fibroblasts treated with TGF-β1, even though the expression of TGF-β is weak or undetectable in the skin [97], suggesting that SSc fibroblasts possess a self-activation system at least partially via autocrine TGF-β signaling. The increased expression of latent TGF-β receptors, including integrin αVβ3, integrin αVβ5 and thrombospondin-1, contributes to this process in SSc fibroblasts [98,99,100,101,102]. These receptors recruit and activate the latent form of TGF-β on the cell surface, and efficiently increase the concentration of active TGF-β around those cells. Thus, dermal fibroblasts are constitutively activated at least by autocrine TGF-β in SSc lesional skin.

SSc dermal fibroblasts have some mechanisms of selectively responding to the pro-fibrotic stimuli of T cells. In normal dermal fibroblasts, collagen production is suppressed by Th1 cells through membrane-associated IFN-γ [103], and by Th2 cells through membrane-associated TNF-α [104], which overcome the pro-fibrotic effect of IL-4. In SSc dermal fibroblasts, by contrast, increased collagen synthesis is resistant to Th1 cell- and Th2 cell-mediated suppression, and especially to the latter [103,104]. A possible mechanism of this property is the secretion of excessive amounts of progranulin, an intrinsic antagonist of TNF receptors, which render SSc dermal fibroblasts resistant to the anti-fibrotic effect of TNF-α in an autocrine and paracrine manner [105]. Considering Th2-skewed immune polarization early in the course of dcSSc, unresponsiveness to Th2 cell-mediated suppression may contribute to fibroblast activation in the early stages of dcSSc.

SSc dermal fibroblasts, vice versa, affect the transdifferentiation of inflammatory cells. For instance, SSc dermal fibroblasts regulate the tissue-localized transdifferentiation of regulatory T cells (Tregs) into Th2-like cells through the IL-33 in the involved skin [58]. Furthermore, SSc dermal fibroblasts suppress the interferon-γ expression of skin-infiltrating CD4PP^+^P T cells through galectin-9 overproduction, promoting skin fibrosis development in the Th2/Th17 predominant microenvironment [106]. Thus, SSc dermal fibroblasts may affect the immune response in the skin much more broadly than previously thought.

Overall, SSc fibroblasts maintain their activated status through the self-activation system and the feed-forward loop by interacting with other cell types, eventually leading to the irreversible fibrotic remodeling of multiple organs.

### 2.6. The Role of Agonistic Autoantibodies

In addition to anti-endothelial cell antibodies and antinuclear antibodies, there is another set of autoantibodies that function as agonists of cell surface receptors. These include antibodies against platelet-derived growth factor receptor (PDGFR), angiotensin II type 1 receptor (AT1R) and endothelin-1 type A receptor (ETaR). The anti-PDGFR antibody induces the collagen production of fibroblasts, and the proliferation and migration of pulmonary vascular smooth muscle cells, possibly contributing to the development of organ fibrosis and PAH [107]. Anti-AT1R and anti-ETaR antibodies are positive in ~85% of SSc patients, and there is a strong positive correlation in the titers of these antibodies [108]. These antibodies induce the expressions of cell adhesion molecules, cytokines and/or chemokines in endothelial cells, B cells, T cells and macrophages [109,110]. Importantly, higher levels of anti-AT1R and anti-ETaR antibodies are associated with severe SSc complications, such as diffuse skin involvement, ILD, PAH, SRC and digital ulcers, and poor prognosis [108,111]. Thus, agonistic autoantibodies seem to directly facilitate the fibrotic and vascular pathogenic processes of SSc.

## 3. The Additional Organ-Specific Pathologies

### 3.1. The Skin

**Figure 3:**
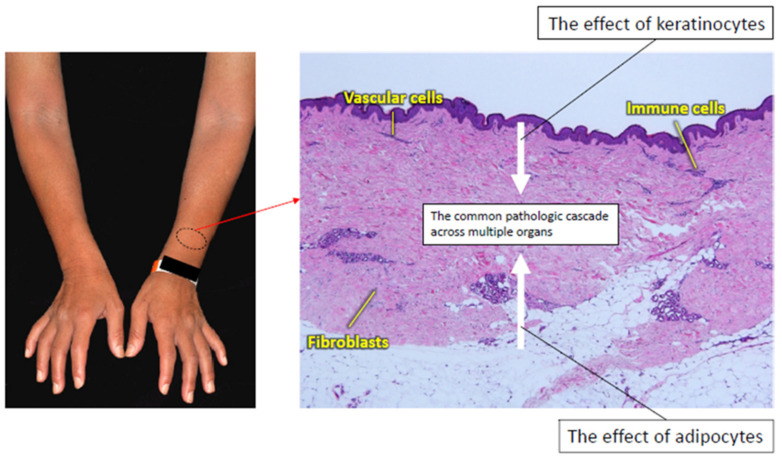
The interaction of the common pathologic cascade with the skin-specific pathology of SSc. The SSc-specific common pathologic cascade is modified by keratinocytes and adipocytes. Keratinocytes produce various factors related to fibroblast activation. Adipocytes serve as a potential source of myofibroblasts by transdifferentiating into mesenchymal cells, and produce a variety of adipokines involved in SSc development.

According to previous studies with clinical samples and animal models, the epidermis and subcutaneous adipose tissue are candidates driving the development of dermal fibrosis in SSc (Figure 3).

#### 3.1.1. Keratinocytes

Over three decades, several studies have demonstrated the upregulation of disease-associated molecules in the epidermis of SSc-involved skin, such as endothelin-1, TGF-β, CCL2, vascular endothelial growth factor, IL-21 receptor, wound healing-associated cytokeratins (keratin 6 and keratin 16), IL-1α, CTGF, IL-6, TNF-α, CCL5, psoriasin and galectin-7 [112,113,114,115,116,117,118,119,120,121]. Considering the potent pro-fibrotic effect of IL-1α, CTGF and IL-6, SSc keratinocytes likely contribute to the activation of dermal fibroblasts.

A potential role of epithelial cells, including keratinocytes, the stratified squamous epithelia of the esophagus and the medullary thymic epithelial cells, in the development of SSc has been proposed in animal experiments with a new SSc murine model [122]. The deficiency of transcription factor Fli1, which is a potential predisposing factor of SSc [7,123], induces SSc-like properties in various types of cells, including fibroblasts, endothelial cells, keratinocytes and macrophages [29,34,122,124,125,126]. Of note, epithelial cell-specific *Fli1* knockout mice (*Fli1*P^flox/flox^P; K14-CreP^+/−^P mice), which possess epithelial cells with SSc-like phenotypical features, spontaneously develop dermal and esophageal fibrosis due to the activation of epithelial cells in the skin and esophagus. Furthermore, these mice exhibit ILD mediated at least by T cells autoreactive to lung antigens, due to impaired negative selection and Treg development in the thymus. A part of the impaired central tolerance is ascribed to the downregulation of autoimmune regulatory (Aire), which regulates the processing and presentation of self-antigens in medullary thymic epithelial cells [127,128]. Importantly, epithelial cell-specific *Fli1* knockout mice without acquired immune systems (*Rag1*P^−/−^P; *Fli1*P^flox/flox^P; *K14*-CreP^+/−^P mice) exhibit the spontaneous development of dermal and esophageal fibrosis, together with mast cell infiltration in the skin, suggesting that the activation of epithelial cells alone can induce tissue fibrosis through the activation of innate immunity. This new murine model suggests that abnormally activated epithelial cells underlie selective organ fibrosis and autoimmunity in SSc.

Another potential component of the keratinocyte-dependent regulation of dermal fibrosis is the crosstalk between the immune system and the skin microbiota. This research area has recently caught much attention as regards inflammatory skin diseases, such as atopic dermatitis [129]. This dialogue is initiated with the sensing of pathogen-associated molecular patterns, derived from microorganisms, through pattern recognition receptors by keratinocytes, followed by the secretion of antimicrobial peptides from those cells, eventually resulting in the death or inactivation of a diverse range of microorganisms and the activation of a variety of immune cells and non-immune cells, including dermal fibroblasts and dermal microvascular endothelial cells [130]. Some antimicrobial peptides are constitutively expressed, while the expression of others is transient and controlled by members of the skin microbiota. So far, the contribution of skin microbiota to the altered phenotype of keratinocytes and skin immunity in SSc remains unclear, though microbiome dysbiosis is reported in SSc-involved skin [131].

#### 3.1.2. Adipocytes

The presence of adjacent and abundant fat tissue is a unique histologic feature of the skin. In the recent decades, the potential contribution of subcutaneous adipose tissue to skin fibrosis has been reported in SSc. As already mentioned, myofibroblasts can originate from non-fibroblast lineage cells residing within the pro-fibrotic microenvironment [132]. According to the lineage-tracing approaches [133], subcutaneous adipocytes are highly plastic cells that can transdifferentiate into myofibroblasts [134]. Indeed, a significant proportion of activated myofibroblasts in SSc-involved skin appear to arise from adipocytes adjacent to the deep dermis [93,135]. On the other hand, adipocytes produce a variety of adipokines with pleiotropic effects on various cell types through autocrine, paracrine and endocrine mechanisms [136]. An altered adipokine balance due to adipocyte loss or dysfunction possibly contributes to the inflammation, vasculopathy and fibrosis characteristic of SSc [137,138,139,140,141,142,143,144,145,146,147]. Among the family of adipokines, the role of adiponectin has been well studied. The serum levels and tissue expression of adiponectin inversely correlate with skin score in SSc patients [137,146,148]. *Adipo*P^−/−^P mice develop less dermal fibrosis in response to bleomycin injection [149], and AdipoRon, a pharmacological inhibitor of adiponectin signaling, suppresses the bleomycin-dependent induction of SSc-like skin fibrosis, vasculopathy and immune abnormalities [150]. Overall, subcutaneous adipose tissue seems to serve as a critical driver of skin fibrosis in SSc.

### 3.2. GI Involvement

GI symptoms are experienced in ~90% of patients with SSc, and are the leading cause of morbidity, characterized by hypomotility, dysmotility and the impaired secretion of digestion enzymes in any GI region from the oral cavity to the anus [151,152,153]. The frequencies of upper and lower gut symptoms are about 70–90% and 20–70%, respectively. The esophagus is the most frequently affected, followed by the anorectal region, the small bowel, stomach and colon. Esophageal dysfunction, including gastroesophageal reflux disease (GERD) and dysphagia, is the most common GI manifestation, while a variety of lower gut symptoms can occur, such as small intestinal bacterial overgrowth, malabsorption, malnutrition, diarrhea, pseudo-obstruction, constipation, pneumatosis intestinalis and fecal incontinence [154].

As is the case with the skin and other internal organs, the common SSc-specific pathologic cascade broadly affects the GI system, eventually resulting in extensive atrophy and fibrosis of the gut smooth muscle [155]. In addition, there is a GI organ-specific pathology of SSc that is relevant to the complex but highly organized enteric nervous system. The vascular structural changes, such as capillary loss and arteriolar stenosis, induce tissue hypoxia throughout the GI tract, resulting in autonomic axonal degeneration [156]. Thus, SSc-associated GI involvement is attributable to hypomotility and dysmotility, due to the extensive atrophy and fibrosis of the enteric smooth muscle and the disturbed enteric nervous system. Indeed, SSc-associated esophageal dysfunction consists of the following three pathological components: (i) reduced lower esophageal sphincter pressure, (ii) ineffective esophageal body peristalsis, especially in the lower part, and (iii) the incoordination of the peristaltic and lower esophageal sphincter function [151,155,157,158,159]. Importantly, esophageal dysfunction often occurs early in the course of SSc, even in the very early cases without skin sclerosis, reflecting impairment to the enteric nervous system due to vasculopathy. On the other hand, esophageal dysmotility progresses rapidly (within years) in dcSSc, while it does so slowly (within decades) in limited cutaneous SSc (lcSSc) [160,161], reflecting the atrophic and fibrotic changes of the gut smooth muscle as a final consequence of SSc-associated GI involvement.

A subset of SSc patients have antibodies against myenteric neurons [162], including an antibody against muscarinic acetylcholine receptor M3 [163,164]. These antibodies interact with myenteric neurons and disrupt intestinal peristalsis in animal models [162,163]. Furthermore, SSc patients with higher titers of the anti-muscarinic acetylcholine receptor M3 antibody show more severe GI phenotypes [165], suggesting that this antibody is pathogenic in humans. Consistent with this notion, the motility of the pharynx and proximal esophagus that is regulated by somatic nerve system is normal in SSc [166]. Overall, the currently available data support the idea that a combination of autoimmunity, vasculopathy and fibrosis underlies the GI involvement associated with SSc.

As described above in the section on “the skin”, squamous stratified epithelia can be a potential driver of esophageal fibrosis. The esophagus of *Fli1*P^flox/flox^P; *K14*-CreP^+/−^P mice represents molecular features of the SSc esophagus, such as the increased expression of IL-8, and it highly expresses IL-1β in its stratified squamous epithelia. Importantly, it is suggested that esophageal epithelium-derived inflammatory cytokines contribute to GERD-related and functional heartburn in patients who continue to experience heartburn symptoms despite adequate-dose proton pump inhibitor therapy [167]. Indeed, several studies have failed to show any correlation between GI symptoms and the severity of GI physiological changes in SSc patients [151,168]. Although further studies are required, the altered phenotype of esophageal epithelium may be involved in the development of esophageal fibrosis and heartburn in SSc patients.

Another important component of the GI-specific pathology is gut microbiota that affect the development and function of the immune system, and seem to play a role in autoimmune diseases through microbiota-related immune dysfunction [169,170,171]. In SSc, several cohorts have demonstrated that intestinal microbiota composition differs from that of healthy individuals; in particular, decreased levels of commensal bacteria, such as *Faecalibacterium*, *Clostridium* and *Bacteroides*, and increased levels of pathobiont bacteria, such as *Fusobacterium* and *γ-Proteobacteria* [172,173]. At this moment, it is unclear whether microbiota changes precipitate and perpetuate the SSc-associated immune system, or result from SSc itself and/or related therapies. Further basic and clinical studies are required to understand the mechanism by which gut microbiota interact with the key inflammatory and fibrotic mediators underlying the development of SSc-specific clinical symptoms.

**Figure 4:**
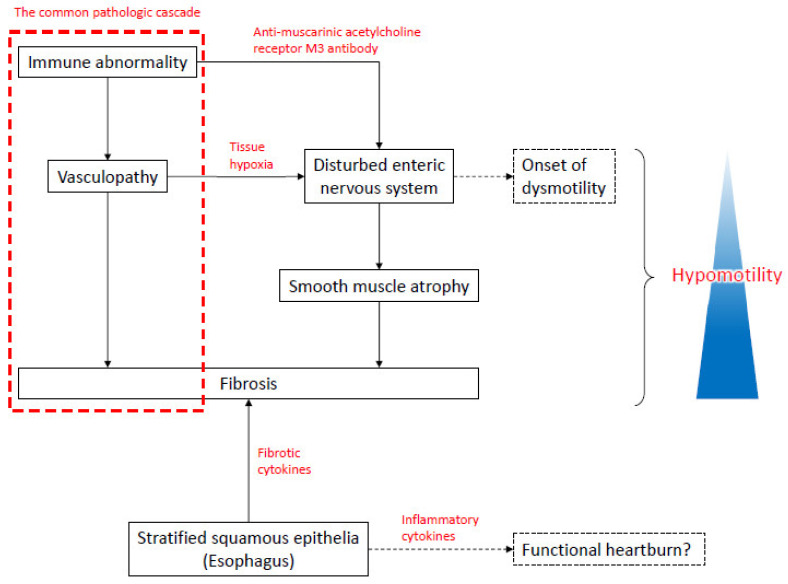
The interaction of the common pathologic cascade with the esophagus-specific pathology in SSc. In addition to the SSc-specific common pathologic cascade, autoimmunity and vasculopathy affect the enteric nerve system through the anti-muscarinic acetylcholine receptor M3 antibody and tissue hypoxia, respectively, leading to dysmotility and hypomotility of the gastrointestinal tract. Persistent hypomotility promotes smooth muscle atrophy and the resultant fibrotic replacement, further contributing to the progression of gastrointestinal tract hypomotility. Esophageal squamous stratified epithelia may facilitate tissue fibrosis and functional heartburn by producing a variety of disease-associated molecules, such as cytokines and chemokines.

Taken together, the abnormally activated stratified squamous epithelia and the dysfunction of the enteric nerve system constitute the organ-specific pathological process of the GI tract in the SSc (Figure 4).

## 4. Lung Involvement

Pulmonary involvement, such as ILD and PH, is the leading cause of SSc-related mortality [174,175]. ILD is caused by the common SSc-specific pathologic cascade, and can be modified by the microaspiration of gastric content due to GERD. SSc-PAH is ascribed to pulmonary arteriolar stenosis due to occlusive vascular fibrosis, while other types of SSc-PH are associated with cardiac involvement and ILD. Indeed, these distinct pathologies may coexist to variable degrees, contributing together to the elevation of pulmonary arterial pressure in patients with SSc-PH.

The World Health Organization (WHO) classification stratifies PH into five categories: group 1, PAH that is characterized by abnormalities of pulmonary arterioles; group 2, PH associated with left heart disease; group 3, PH associated with lung disease/hypoxia; group 4, PH due to pulmonary embolus or thrombosis, also referred to as chronic thromboembolic pulmonary hypertension (CTEPH); and group 5, PH due to other miscellaneous causes, which do not fit into the other four categories. SSc-PH belongs to group 1, 2 or 3, and SSc patients complicated with anti-phospholipid antibody syndrome may develop group 4 PH. SSc-PAH is more common in patients with lcSSc and in those with the anticentromere antibody (ACA) [176]. SSc-PAH is histologically characterized by the fibrotic occlusion of pulmonary arterioles, which occurs as a result of the common SSc-specific pathologic cascade. SSc-PAH frequently accompanies the histologic changes of pulmonary venules that are similar to those of pulmonary veno-occlusive disease (PVOD) [177,178].

ILD is detected in 50–60% of SSc patients by high resolution computed tomography [179,180]. The risk factors for the development of SSc-ILD include dcSSc [181], African American ethnicity [182], shorter disease duration [183], older age at disease onset [181], and the presence of anti-topo I antibody and/or absence of ACA [181]. ILD typically occurs early in the course of dcSSc, especially within 3 years after the onset of disease [181,183,184], whereas ILD occurs at any time of disease course in lcSSc patients [185]. The clinical course of SSc-ILD is variable; some patients show stability in forced vital capacity (FVC), while others show a progressive decline in lung function [186]. ILD mostly progresses within 4 years after the onset of SSc, and afterward the progression becomes slow or stops completely, even without any treatment [187]. It is reported that severe ILD, showing a decline in FVC below 50%, constitutes around 15% of total SSc [187,188]. The histological pattern most commonly observed in SSc-ILD is nonspecific interstitial pneumonia (NSIP), which is observed in approximately two-thirds of patients [189]. Usual interstitial pneumonia (UIP) is present in a minority of individuals with SSc-ILD [189,190,191], and may be associated with poorer outcomes [192].

Histologically, NSIP associated with SSc-ILD is stratified into four stages [193]. The initial stage is characterized by the overdevelopment of microvessels with abnormal structures, as well as the thickening of the alveolar septa with a large number of α-SMA-positive myofibroblasts. The overdeveloped microvessels contain blood cells in their lumina, suggesting that a functional circulation is maintained. In the second stage, the degree of ECM deposition is substantial and progressive, with the septal borders of alveoli being irregular and obscure. The microvessels are further structurally disorganized, and larger blood vessels are obliterated. In addition, the disarray or partial loss of the alveolar epithelium is evident. In the third stage, the progression of fibrosis extensively damages the vital structures of the lung, including alveoli and vasculature. In the final stage, the lung transforms into a contracted fibrous organ lacking alveoli and vasculature. The initial changes of the microvessels and the subsequent progressive fibrous changes support the notion that SSc-ILD is mediated by the common SSc-specific pathologic cascade, as is the case with other organs.

A potential driver associated with the progression of SSc-ILD is the microaspiration of gastric content due to GERD. This notion is supported by the clinical data, histologic analysis and animal experiments. Several clinical studies have demonstrated the positive correlation of an increased degree of lung fibrosis with more frequent reflux episodes and a greater proximal extent of refluxate [194]. In a rat model of GERD, parenchymal fibrosis can be induced by gastric content introduced into the lung [195]. The analysis of lung biopsy samples identified a novel histological form of lung disease, known as centrilobular fibrosis, which is seen especially in SSc patients with severe GERD [196]. Centrilobular fibrosis is characterized by the predominant bronchocentric distribution of the lesions, and the presence of intraluminal basophilic content and foreign bodies inside the bronchi, occasionally with multinucleated giant cell reactions. In a previous study with open lung biopsies obtained from 22 patients with SSc-ILD [197], 21% of cases showed CLF alone (isolated CLF), and 84% of patients with a predominant NSIP pattern had a CLF pattern, suggesting that GERD may exacerbate the underlying NSIP in SSc-ILD. Although any trial data have not revealed an improvement in the pulmonary function of SSc-ILD after aggressive GERD management, it is possible that the majority of SSc patients would benefit from aggressive GERD treatment. Overall, GERD is an organ-specific disease modifier of SSc-ILD.

## 5. Heart

According to the histologic examination of autopsy tissues from SSc patients with no cardiac symptoms before death, all the patients display evidence of myocardial disease [198]. Thus, cardiac involvement occurs in almost all the SSc patients, but it is often clinically occult [199,200]. Once clinically apparent, however, cardiac involvement has a very poor prognosis [199,201,202,203]. There is a wide range of clinical manifestations related to primary cardiac involvement in SSc, including arrhythmias, conduction system defects, myocarditis, pericarditis, systolic and diastolic ventricular dysfunction, and heart failure [204,205]. Primary myocardial involvement accounts for ~30% of deaths in patients with SSc [175,203,206].

Although the molecular mechanism of SSc-related cardiomyopathy remains unclear [204,207,208,209], a canonical wisdom is that microvascular disease plays a central role; that is, the vascular structural abnormalities, such as capillary loss and arteriolar stenosis, induce tissue hypoxia, subsequently promoting inflammation and the production of excessive ECM by cardiac fibroblasts [199]. Indeed, the histologic evaluation of myocardial specimens from SSc autopsy subjects demonstrated inflammation, vascular changes and ECM deposition greater than those observed in specimens from age- and sex-matched control autopsy subjects [198]. Supporting the critical role of microvasculature, but not coronary arteries, fibrotic changes in the myocardium are often patchy and distributed over both ventricles, independent of coronary artery supply territories [207,210].

The abnormal vasoreactivity of small arteries and arterioles, what we call “myocardial Raynaud’s phenomenon”, plays a part in SSc-associated cardiac involvement. This notion is supported by evidence that the absence of previous treatment with calcium channel blockers is an independent factor associated with left ventricular dysfunction. Furthermore, vasodilators, such as nifedipine, nicardipine and captopril, mitigate acutely both myocardial perfusion and function [211,212,213,214], possibly through the lowering of myocardial vascular resistance. More importantly, ~30% of SSc patients with long-history Raynaud’s phenomena show myocardial Raynaud’s phenomena after exposure to cold air, which is inhibited by the administration of calcium channel blockers [215].

Overall, from the perspective of management, it is quite important to know that two distinct vascular changes, such as structural changes (capillary loss and arteriolar stenosis) and functional abnormality (myocardial Raynaud’s phenomenon), contribute to the cardiac involvement of SSc.

## 6. Liver

SSc is rarely associated with severe liver complications. SSc patients with ACA are frequently positive for antimitochondrial M2 antibody, but the clinical manifestation of primary biliary cholangitis is so mild that no treatment or oral intake of ursodeoxycholic acid is selected [216,217,218]. Histologically, the liver of SSc patients represents portal tract fibrosis even in the absence of any other abnormalities [219]. However, portal hypertension is quite rare in SSc patients.

A differential diagnosis of portal hypertension associated with SSc is nodular regenerative hyperplasia of the liver (NRHL), which is histologically defined by diffuse micronodular transformation without fibrous septa. NRHL is thought to develop as a result of microvascular alterations due to endothelial cell damage. Patients with NRHL may remain asymptomatic, but at least 50% of reported cases represent portal hypertension and related symptoms, including splenomegaly, ascites and esophageal or gastric varices. Transaminases might be normal or slightly elevated, whereas cholestatic measures are often more significantly increased [220,221]. Among autoimmune disorders, SSc in particular has been suggested to be associated with NRHL. SSc-associated NRHL generally occurs in patients with ACA [222]. Given that vascular injury is an initial trigger of the SSc-specific disease cascade, SSc-associated vascular injury may promote the development of NRHL in certain individuals highly susceptible to this complication. Supporting the contribution of vasculopathy to the development of NRHL, SSc patients with NHRL are highly susceptible to vascular complications, such as digital ulcers, PAH and SRC.

The liver is relatively protected from extensive fibrosis relative to other organs, such as the skin, GI tract, lungs and heart. So far, the underlying mechanism remains unknow, but the unique vascular system of the liver may modify the common pathologic cascade of SSc, especially the process bridging vasculopathy and tissue fibrosis.

## 7. SRC

Up to 10% of SSc patients are complicated with SRC, which is most frequently seen in dcSSc, especially within the first 3 years after the onset [223,224]. The anti-RNAP III antibody is significantly associated with the development of SRC [225,226,227], and an association with the anti-topo I antibody in dcSSc patients has also been reported [228]. The rapid progression of skin sclerosis, frequently seen in patients with antibodies against RNAP III and topo I antigens, and high doses of corticosteroid therapy are risk factors for this complication [228,229]. Although still controversial or preliminary, calcium channel blockers reeuce the risk of SRC [230], while angiotensin converting enzyme inhibitors prior to the onset of SRC worsen its prognosis [231].

SRC is typically characterized by a sudden and marked increase in systemic blood pressure and acute renal failure, with or without significant microangiopathic hemolytic anemia or thrombocytopenia. SRC is often accompanied by headache, blurring of vision, and dyspnea due to hypertensive encephalopathy, congestive heart failure and/or pulmonary edema [232,233]. There is a subtype of SRC with normal or mildly elevated blood pressure, namely, normotensive SRC [234], the prognosis of which is relatively poor due to delayed diagnosis.

SRC seems to be caused by the common pathologic cascade of SSc. SRC is histologically characterized by early vascular changes, such as the intimal accumulation of myxoid material, thrombosis, and/or fibrinoid necrosis of glomerular capillaries [235], suggesting that it is initiated by a rapid increase in endothelial permeability and intimal edema, leading to the direct contact of circulating blood elements with the subendothelial connective tissue, the activation of the coagulation cascade and the resultant vascular thrombosis. The vascular injury and related inflammation promote fibroblastic and non-fibroblastic stromal proliferation, that is, proliferative obliterative vasculopathy (onion skin type lesion). A decrease in renal perfusion induces juxtaglomerular apparatus hyperplasia and renin secretion, resulting in accelerated hypertension and progressive renal injury. The critical rise in systemic blood pressure causes further damage to renal blood vessels and initiates a feedforward cycle, eventually leading to malignant hypertension [236,237,238]. A milder form of vascular pathology, such as fibrointimal thickening, is often observed in SSc patients without SRC, and to a greater extent in dcSSc relative to lcSSc [239]. Apart from other organ involvement, the fibrosis is usually restricted to perivascular regions.

Although the entire pathogenesis of SRC remains unknown, renal vasospasm (Raynaud’s phenomenon) likely exacerbates renal ischemia, together with structural changes in the blood vessels (intimal thickening and proliferation, fibrin deposition) [240]. This notion is supported by the potential preventive effect of calcium channel blockers on the development of SRC [230].

Overall, the pathogenesis of SRC should be understood based on two distinct vascular changes, namely, structural changes (capillary loss and arteriolar stenosis) and functional abnormality (renal vasospasm), as is the case with heart involvement.

## 8. Conclusions

SSc manifests with a broad scope of organ involvement related to vasculopathy and tissue fibrosis. Although extremely complicated, the pathogenesis of SSc can be categorized into the common pathologic cascade across multiple organs and the additional organ-specific pathologies. From the perspectives of disease management and generating new therapies, it is quite important to understand the differences and interactions between these two distinct disease components underlying the development of SSc.
